# Association between the healthy eating index 2020 and heart failure among the U.S. middle-aged and older adults from NHANES 2005–2020: a cross-sectional study

**DOI:** 10.3389/fnut.2024.1496379

**Published:** 2025-01-06

**Authors:** Fangfang Gu, Weiwei Yu, Tian Shu, Yingwei Zhu

**Affiliations:** Department of Cardiology, Huzhou Central Hospital, Fifth School of Clinical Medicine of Zhejiang Chinese Medical University, Huzhou, Zhejiang, China

**Keywords:** HEI-2020, HF, middle-aged and older adults, NHANES, cross-sectional study

## Abstract

**Objective:**

This study aims to shed light on the correlation between Healthy Eating Index-2020 (HEI-2020) and heart failure (HF) in American adults aged 50 or above.

**Methods:**

Data were from the National Health and Nutrition Examination Survey 2005–2020, encompassing 13,105 participants with an age of 50 or above. HEI-2020 score was utilized for rating the dietary quality. The link of HEI-2020 to HF was assessed via logistic regression, restricted cubic splines (RCS), generalized additive models (GAM), weighted quantile sum (WQS) regression, as well as quantile g-computation (Qgcomp) models.

**Results:**

A negative association between HEI-2020 and HF risk was uncovered in middle-aged and older Americans (*OR* = 0.99, *95% CI*: 0.98–1.00, *p* = 0.006). The highest quartile (Q4) exhibited a markedly lower HF risk than the lowest quartile (Q1) (*OR* = 0.70, *95% CI*: 0.55–0.89, *p* = 0.004). RCS and GAM analyses demonstrated a linear dose–response relationship between HEI-2020 and HF. Finally, WQS regression and Qgcomp models revealed a beneficial combined influence of 13 dietary components on HF risk, with dairy and whole fruits emerging as the most influential.

**Conclusion:**

Elevated HEI-2020 scores are linked to decreased HF risks among Americans aged 50 or above, suggesting that adherence to the Dietary Guidelines for Americans can mitigate HF risk.

## 1 Introduction

Heart failure (HF), a prevalent and intricate illness, often arises from weakened left ventricular myocardial function. It manifests as dyspnea, fatigue, less exercise tolerance, fluid retention, pulmonary edema, and peripheral edema (1). It is estimated that over 60 million individuals globally are afflicted with HF, and its prevalence will continue rising as global people age (2, 3). Furthermore, the mortality attributed to HF is on the rise, imposing a heavy social and economic burden on society (4).

In recent years, drug, surgical, and other interventions have markedly improved HF patients’ survival and well-being (5–8). Medications including angiotensin-converting enzyme and angiotensin receptor-neprilysin inhibitors, *β*-blockers, and mineralocorticoid receptor antagonists, could significantly lower all-cause and cardiovascular mortality, as well as all-cause and HF-related hospitalization rates among HF sufferers (5). Despite advancements in drug therapy, the mortality remains high among patients with advanced HF. Established treatments for advance HF include mechanical circulatory support, valve repair or replacement, coronary artery bypass grafting, and heart transplantation (9, 10). Additionally, non-pharmacological treatments, such as exercise (6), acupuncture (7), and massage therapy (8) can ameliorate the quality of life for HF patients. Overweight and obesity contribute to increased HF risk through mechanisms like neurohormonal activation, adipose tissue effects, hemodynamic changes, ectopic fat deposition, and fat toxicity (11). Kehagias et al. (12) demonstrated that sleeve gastrectomy promotes sustained weight loss and improves cardiovascular conditions, such as hypertension, diabetes, and dyslipidemia. While these therapies are important in HF management, the effects of diet on HF prevention and treatment warrants careful consideration (13, 14). Wickman et al. (13) proved that the Dietary Approaches to Stop Hypertension (DASH) diet exerts positive influence on HF patients. Additionally, Ibsen DB et al. revealed that long-term DASH diet or substitution of DASH-related foods can guard against the progression of HF (15). Recently, more studies have delved into the connection of diet with chronic conditions, such as cognitive, metabolic, pulmonary, and cardiovascular diseases (16–18). However, few have examined the specific interplay between diet and HF. Healthy Eating Index (HEI), a quantitative indicator for evaluating the dietary quality of American population, was developed by the National Cancer Institute (NCI) under the U.S. Department of Health and Human Services, in collaboration with the Center for Nutrition Policy and Promotion under the U.S. Department of Agriculture (USDA) (19–21). It is updated with the Dietary Guidelines for Americans (DGA) and has undergone revisions in 2005, 2010, 2015, and 2020. The 2020 version is the latest and currently under study (22).

We seek to clarify the possible relationship between HEI-2020 and HF in Americans aged 50 or above based on 2005–2020 data of the National Health and Nutrition Examination Survey (NHANES). In view of relatively high prevalence and mortality of HF in the U.S., combined with the significant effects of diet on health, this research holds substantial significance for public health.

## 2 Methods

### 2.1 Study sample

**Figure 1:**
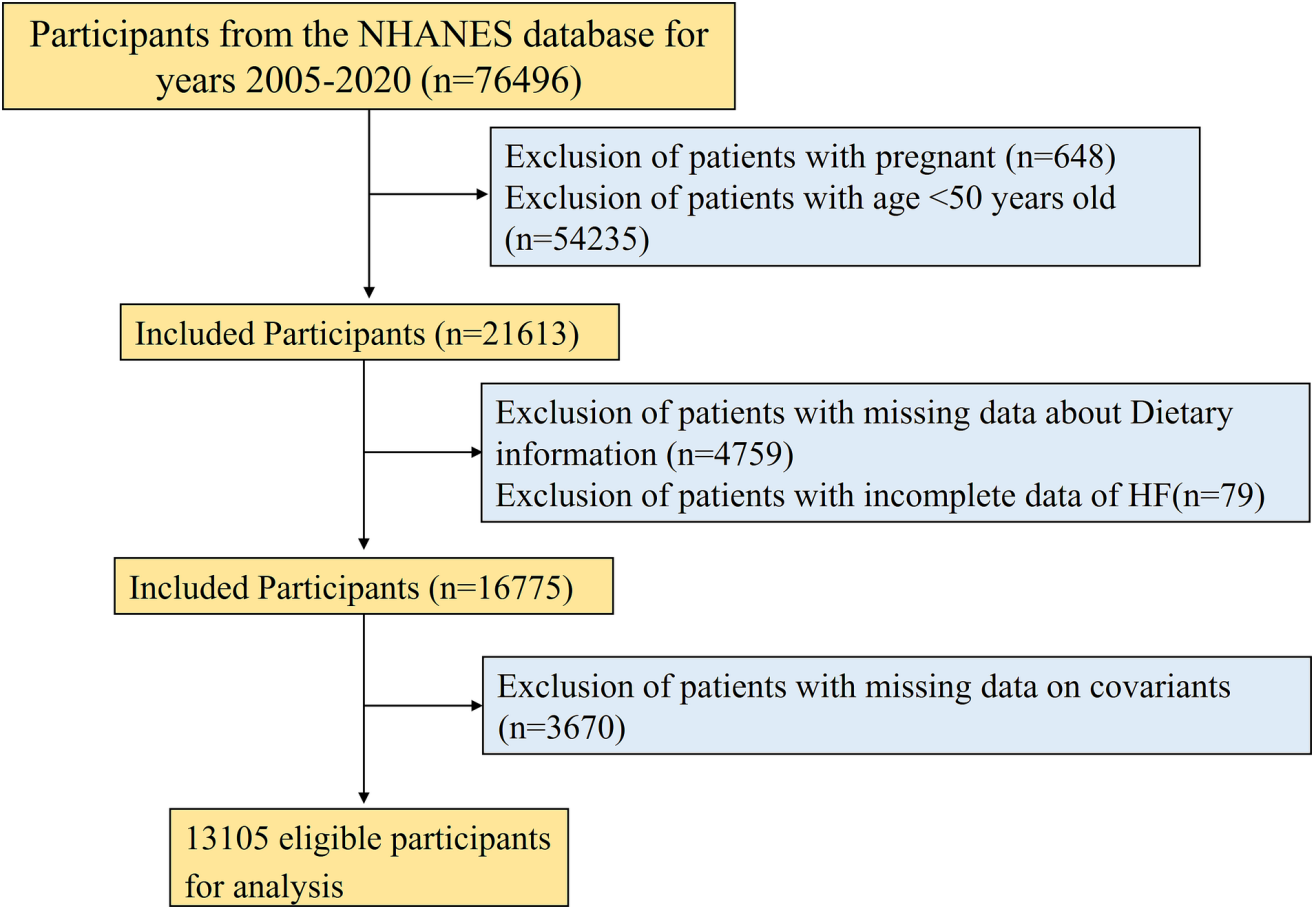
Flow chart.

NHANES, a large-scale cross-sectional study carried out biennially by the National Center for Health Statistics (NCHS) of the U.S. Centers for Disease Control and Prevention, is accessible at https://www.cdc.gov/nchs/nhanes/index.htm (23). Its primary aim is monitoring the nutritional intake and health condition of non-institutionalized American residents and serves as a reference for public health policies. To ensure national representativeness, it employs a sophisticated, multistage sampling design. A total of 76,496 participants were included from seven consecutive cycles of NHANES data from 2005 to 2020. To guarantee that our results are complete and reliable, specific exclusion criteria were applied: (1) pregnant women (*n* = 648); (2) participants aged <50 years (*n* = 54,235); (3) participants without HEI-2020 index (*n* = 4,759); (4) participants with missing HF assessment (*n* = 79); (5) participants without incomplete covariate records (*n* = 3,670). Ultimately, 13,105 participants were included. A flowchart for the screening process is presented in Figure 1. The baseline characteristics of both the excluded and included participants were presented in Supplementary Table S1.

### 2.2 Dietary quality

HEI-2020, a dietary quality index established by the USDA Center for Nutrition Policy and Promotion in adherence to the 2020–2025 DGA with factors and scoring criteria identical to those in HEI-2015, comprises 13 different components: total vegetables, greens and beans, total fruits, whole fruits, whole grains, dairy, total protein foods, seafood and plant proteins, fatty acids, sodium, refined grains, saturated fats, and added sugars (22). HEI-2020 score is from 0 to 100, with a higher score reflecting a healthier diet (24). The scores were classified into Q1 (reference group), Q2, Q3, and Q4 (25). During review from 2005 to 2020, the dietary assessment methods used by the NHANES were relatively consistent. Participants were asked to recall and record all foods and beverages they had in the past 24 h, providing detailed information such as food descriptions, quantities, and timing. Compared to other diet evaluation approaches, this 24-h dietary recall method has several advantages: (1) It captures comprehensive details about participants’ daily diets, including various foods and dietary consumption patterns; (2) It can be adapted for use with other health assessment indicators; (3) The use of the 24-h recall method yields more scientifically rigorous results and is widely employed. However, it also has drawbacks, including recall bias resulting from inaccuracies in memory or omissions, and its inability to capture long-term dietary patterns, as it only provides short-term dietary information.

### 2.3 HF diagnosis

The HF was determined based on answers to a household interview question (“Have you been diagnosed with congestive heart failure?”). A positive response was deemed indicative of HF (26, 27).

### 2.4 Covariates

To minimize the impact of confounders and draw sound conclusions, we selected demographic characteristics, behaviors, and chronic diseases as covariates based on relevant literature (18, 28). The demographic covariates encompassed age, gender (male/female), race (Mexican American, Other Hispanic, Non-Hispanic White, Non-Hispanic Black, Other Race), education level (less than high school, high school or equivalent, college or above), marital status (married/living with partner, widowed/divorced/separated, never married), and poverty-to-income ratio (PIR) (low income [<1], medium income [1–3], high income [≥3]). Behavioral covariates included smoking (never/former/current smoker), drinking (never/former/mild/moderate/heavy drinker), and body mass index (BMI). Chronic diseases included diabetes, hypertension, hyperlipidemia, and coronary heart disease.

### 2.5 Statistical analysis

As per NHANES analysis guidelines, the calculations were adjusted for unequal selection probabilities, subgroup oversampling, and non-response in the analyses with sampling weights, strata, and primary sampling units. Categorical variables were expressed as counts and percentages, continuous variables in normal distribution as mean ± standard deviation (SD), and those with non-normal distribution as median and interquartile range (IQR). Baseline characteristics were compared across groups via *t*-test, chi-square (*X*^2^) and Mann–Whitney *U* tests. Next, a weighted logistic regression model assisted in assessing the link of HEI-2020 scores to HF in adults during their middle and later life stages with results in odds ratios (OR) and 95% confidence intervals (CI). HEI-2020 scores were stratified into four quartiles, and the correlation of HEI-2020 scores with HF among the middle-aged and older individuals were elucidated through logistic regression. Restricted cubic splines (RCS) were employed for exploring the dose–response relationship between HEI-2020 scores and HF, with the findings further validated through the generalized additive model (GAM) regression (29). Finally, through a weighted quantile sum (WQS) regression model (30), the combined exposure effects of 13 dietary components in HEI-2020 and health contribution ratio of each component were investigated. The combined and independent effects of the scores on HF were evaluated, and the WQS results were verified via a quantile g-computation (Qgcomp) model (27).

Every statistical test was two-sided, and *p* < 0.05 signified statistical significance. R 4.3.3^1^ was employed for statistical analysis.

## 3 Results

### 3.1 Baseline characteristics of the study population

**Figure 2:**
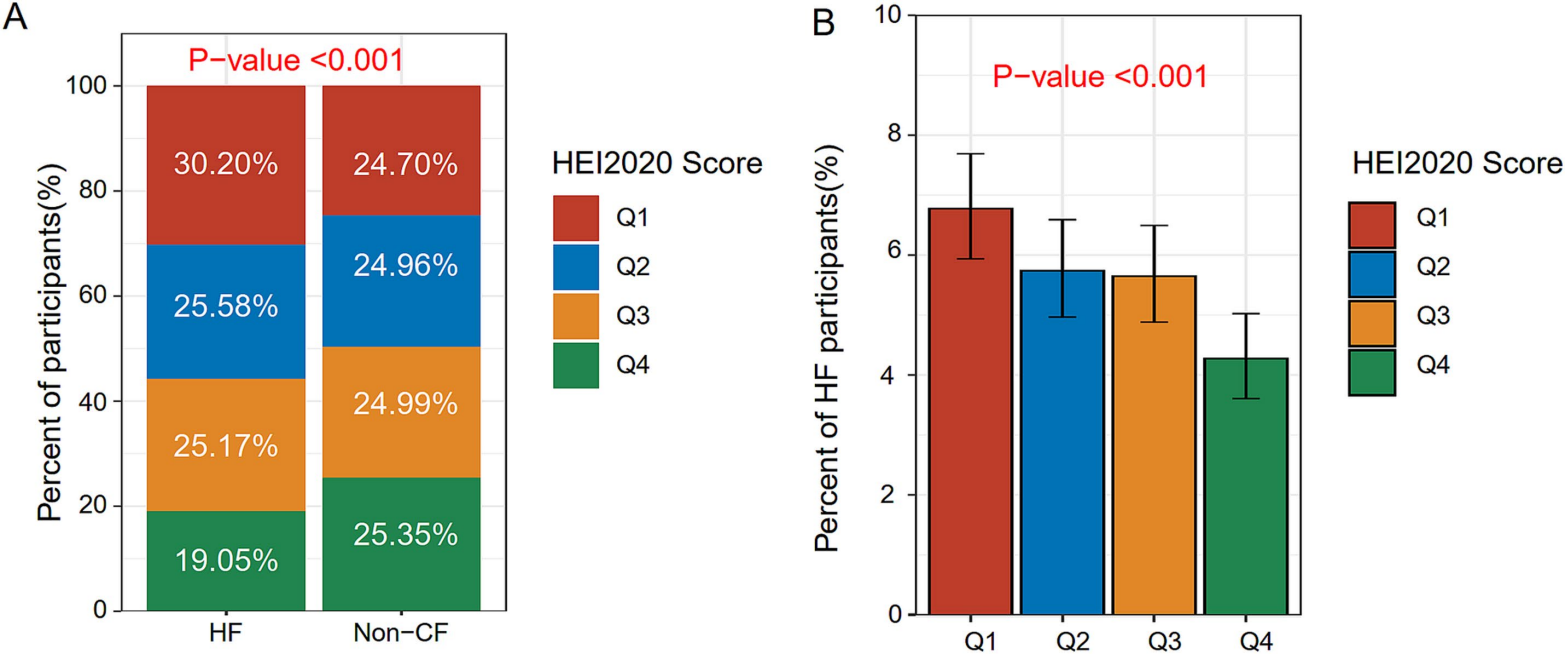
Distribution of HEI-2020 scores and HF across different populations. **(A)** Distribution of HEI-2020 scores between the non-HF group and the HF group. **(B)** Prevalence of HF across different HEI-2020 quartile groups.

After sample screening, 13,105 participants were included, comprising 735 HF patients and 12,370 non-HF individuals. The mean HEI-2020 score was 53.65 ± 12.08, as detailed in Table 1. In contrast to the non-HF cohort, HF patients tended to be older, male, former smokers or drinkers, divorced, separated, or widowed, and have a higher BMI, lower education levels (high school or below), lower income, and a higher diabetes and coronary heart disease prevalence. HEI-2020 scores of HF patients were notably decreased compared with non-HF participants (*p* < 0.001), with a larger proportion in Q1 (29.70%), as depicted in Figure 2A. We also stratified the population into Q1 (<44.86), Q2 (≥44.86 and <52.97), Q3 (≥52.97 and <61.73), and Q4 (≥61.73) based on HEI-2020 scores, as presented in Supplementary Table S2. Compared with those in lower HEI-2020 quartiles, individuals in the higher quartile (Q4) tended to be older, female, non-Hispanic White, married or living with partner, never smokers, moderate drinkers, and have a college education or above, and more income. The incidence of HF decreased progressively across Q2, Q3, and Q4 in comparison to Q1, with significant differences observed (*p* < 0.001), as indicated in Figure 2B.

**Table 1:** The population characteristics among Americans by heart failure.

Characteristics (weighted%)^a^	Total (*n* = 13,105)	HF (*n* = 735)	No-HF (*n* = 12,370)	*p* ^b^
Median Age (IQR), years	64.00 [57.00, 72.00]	70.00 [62.00, 78.00]	63.00 [56.00, 72.00]	<0.001
Gender (*n*/%)				<0.001
Male	6,305 (45.61%)	426 (52.70%)	5,879 (45.29%)	
Female	6,800 (54.39%)	309 (47.30%)	6,491 (54.71%)	
Race (*n*/%)				<0.001
Mexican American	1,563 (4.15%)	50 (2.73%)	1,513 (4.21%)	
Other Hispanic	1,188 (3.68%)	47 (2.46%)	1,141 (3.74%)	
Non-Hispanic White	6,546 (78.21%)	419 (78.32%)	6,127 (78.20%)	
Non-Hispanic Black	2,872 (9.06%)	192 (13.30%)	2,680 (8.87%)	
Other-Race	936 (4.90%)	27 (3.19%)	909 (4.98%)	
Education (*n*/%)				<0.001
High school or below	3,262 (14.81%)	239 (26.77%)	3,023 (14.27%)	
High school or equivalent	3,164 (24.97%)	208 (30.74%)	2,956 (24.70%)	
College or above	6,679 (60.22%)	288 (42.49%)	6,391 (61.03%)	
Marital status (*n*/%)				<0.001
Married or living with partner	7,881 (66.39%)	385(56.63%)	7,496 (66.83%)	
Divorced, separated, or widowed	4,327 (27.79%)	305 (37.91%)	4,022 (27.33%)	
Never married	897 (5.82%)	45 (5.46%)	852 (5.84%)	
PIR (*n*/%)				<0.001
Low income (PIR < 1)	2,161 (9.35%)	170 (16.28%)	1991 (9.03%)	
Medium income (PIR ≥ 1 and PIR < 3)	5,621 (34.61%)	398 (53.30%)	5,223 (33.75%)	
High income (PIR ≥ 3)	5,323 (56.05%)	167 (30.42%)	5,156 (57.22%)	
Smoking status (*n*/%)				<0.001
Never smoker	6,925 (54.16%)	321 (44.79%)	6,604 (54.59%)	
Former smoker	4,065 (30.63%)	277 (37.24%)	3,788 (30.32%)	
Current smoker	2,115 (15.21%)	137 (17.97%)	1978 (15.09%)	
Drinking status (*n*/%)				<0.001
Never drinker	3,008 (18.21%)	194 (25.35%)	2,814 (17.88%)	
Former drinker	2,334 (14.92%)	208 (26.47%)	2,126 (14.39%)	
Mild drinker	7,032 (59.79%)	306 (44.05%)	6,726 (60.51%)	
Moderate drinker	331 (3.06%)	10 (1.35%)	321 (3.14%)	
Heavy drinker	400 (4.03%)	17 (2.78%)	383 (4.09%)	
BMI (mean ± SD)	29.6 ± 6.65	31.83 ± 8.05	29.47 ± 6.54	<0.001
Diabetes (*n*/%)	301 (1.76%)	49 (5.61%)	252 (1.58%)	<0.001
Hypertension (*n*/%)	1867 (13.03%)	106 (13.36%)	1761 (13.01%)	0.932
Hyperlipidemia (*n*/%)	1,073 (7.27%)	62 (8.31%)	1,011 (7.22%)	0.855
Coronary heart disease (*n*/%)	989 (6.83%)	311 (43.24%)	678 (5.16%)	<0.001
HEI-2020 (mean ± SD)	53.65 ± 12.08	51.64 ± 11.46	53.77 ± 12.11	<0.001

^aThe number of participants is unweighted. All percentage estimates are weighted.^

^bp-value was based on χ2 or analysis of variance or Kruskal–Wallis rank sum test where appropriate.^

### 3.2 Association between HEI-2020 and HF

The correlation of HEI-2020 scores with HF risk in adults during their middle and later stages of life (aged 50 or above) was analyzed through logistic regression models, with the results detailed in Table 2. In Model 3, an adverse link was identified between the continuous HEI-2020 variable and HF risk in this cohort (*OR* = 0.99, *95% CI*: 0.98–1.00, *p* = 0.006). Moreover, participants in the highest HEI-2020 quartile (Q4) exhibited an obviously lower risk of developing HF than those in the lowest HEI-2020 quartile (Q1) (*OR* = 0.70, *95% CI*: 0.55–0.89, *p* = 0.006).

**Table 2:** Association between HEI-2020 and HF in middle-aged and older adults aged 50 or above.

Variable	Model 1		Model 2		Model 3	
	OR (95%CI)	*p*	OR (95%CI)	*p*	OR (95%CI)	*p*
HEI-2020 continuous	0.99 (0.98,0.99)	<0.001	0.99 (0.98,0.99)	<0.001	0.99 (0.98,1.00)	0.006
Q1 (<44.86)	–	–	–	–	–	–
Q2 (≥44.86 and <52.97)	0.84 (0.69,1.02)	0.084	0.85 (0.69,1.04)	0.113	0.88 (0.71,1.10)	0.253
Q3 (≥52.97 and <61.73)	0.82 (0.67,1.01)	0.059	0.84 (0.68,1.03)	0.102	0.87 (0.69,1.08)	0.206
Q4 (≥61.73)	0.61 (0.49,0.76)	<0.001	0.64 (0.51,0.81)	<0.001	0.70 (0.55,0.89)	0.004
*P* for trend		<0.001		<0.001		0.006

Model 1: unadjusted.

Model 2: adjusted for age, gender, race, PIR, marital status, education.

Model 3: adjusted for those included in Model 2 as well as BMI, smoking status, drinking status, diabetes, hypertension, hyperlipidemia, and coronary heart disease.

### 3.3 Subgroup analysis and interaction of the association between HEI-2020 and HF

We performed subgroup analyses and interaction assessments based on age, gender, race, marital status, education, smoking, drinking, diabetes, and coronary heart disease, with the results shown in Table 3. HEI-2020 score demonstrated a negative association with HF in middle-aged and older individuals, with the trend primarily observed in specific subgroups, including those aged 50–65, individuals of other races, current smokers, and those without diabetes or coronary heart disease. However, this association was not observed in other subgroups. Moreover, evident interaction effects were found for smoking status (*P for interaction* = 0.021) and diabetes (*P for interaction* = 0.037), indicating that the adverse connection of HEI-2020 with HF was deemed significant only for the current smokers and non-diabetic subgroups.

**Table 3:** Association between HEI-2020 and HF in demographic subgroups.

Characteristics	*N* (%)	Q1 (<44.86)	Q2 (≥44.86 and <52.97)	Q3 (≥52.97 and <61.73)	Q4 (≥61.73)	*P* for trend	*P* for interaction
		OR (95%CI)	OR (95%CI)	OR (95%CI)	OR (95%CI)		
Age group (*n*/%)							0.188
>65	5,738 (43.8%)	Ref	0.89 (0.63,1.28)	0.97 (0.68,1.39)	0.73 (0.51,1.02)	0.508	
50–65	7,367 (56.2%)	Ref	0.93 (0.62,1.41)	0.53 (0.31,0.89)	0.43 (0.22,0.86)	<0.001	
Gender							0.897
Male	6,305 (48.1%)	Ref	0.85 (0.59,1.22)	0.87 (0.65,1.16)	0.75 (0.49,1.15)	0.099	
Female	6,800 (51.9%)	Ref	1.16 (0.78,1.73)	1.03 (0.65,1.63)	0.72 (0.48,1.07)	0. 150	
Race (*n*/%)							0.105
Mexican American	1,563 (11.9%)	Ref	0.57 (0.25,1.30)	0.75 (0.29,1.90)	0.66 (0.24,1.78)	0.171	
Other Hispanic	1,188 (9.1%)	Ref	0.86 (0.33,2.21)	0.57 (0.21,1.58)	0.50 (0.16,1.53)	0.110	
Non-Hispanic White	6,546 (50%)	Ref	1.05 (0.77,1.43)	1.01 (0.71,1.44)	0.80 (0.56,1.13)	0.490	
Non-Hispanic Black	2,872 (21.9%)	Ref	0.63 (0.42,0.95)	0.80 (0.55,1.18)	0.68 (0.40,1.16)	0.321	
Other Race	936 (7.1%)	Ref	2.01 (0.61,6.60)	0.54 (0.18,1.60)	0.20 (0.06,0.66)	0.003	
Marital status (*n*/%)							0.198
Married/Living with partner	7,881 (60.1%)	Ref	0.95 (0.68,1.33)	1.01 (0.70,1.46)	0.66 (0.43,1.03)	0.134	
Widowed/Divorced/Separated	4,327 (33%)	Ref	1.20 (0.8,1.80)	0.90 (0.62,1.32)	0.98 (0.67,1.43)	0.079	
Never married	897 (6.8%)	Ref	0.49 (0.21,1.12)	0.56 (0.11,2.89)	0.23 (0.05,1.10)	0. 301	
Education (*n*/%)							0.846
High school or below	3,262 (24.9%)	Ref	0.90 (0.58,1.39)	1.01 (0.65,1.56)	0.66 (0.41,1.08)	0.193	
High school or equivalent	3,164 (24.1%)	Ref	0.91 (0.56,1.47)	1.36 (0.82,2.25)	1.25 (0.68,2.30)	0.599	
College or above	6,679 (51%)	Ref	1.14 (0.76,1.73)	0.81 (0.52,1.26)	0.71 (0.45,1.12)	0.105	
Smoking status (*n*/%)							0.021
Never smoker	6,925 (52.8%)	Ref	1.04 (0.72,1.51)	1.03(0.67,1.60)	0.83(0.50,1.37)	0. 404	
Former smoker	4,065 (31%)	Ref	0.84 (0.58,1.22)	0.87(0.52,1.46)	0.75(0.49,1.14)	0.450	
Current smoker	2,115 (16.1%)	Ref	1.12 (0.63,1.98)	0.91(0.48,1.71)	0.23(0.09,0.58)	0.033	
Drinking status (*n*/%)							0.540
Never drinker	3,008 (23%)	Ref	1.15 (0.66,2.00)	1.30(0.81,2.07)	0.95(0.57,1.58)	0.215	
Former drinker	2,334 (17.8%)	Ref	1.21 (0.77,1.91)	0.90(0.56,1.45)	0.72(0.42,1.24)	0.173	
Mild drinker	7,032 (53.7%)	Ref	0.81 (0.55,1.19)	0.81(0.49,1.32)	0.67(0.42,1.06)	0.053	
Moderate drinker	331 (2.5%)	Ref	1.77 (0.19,16.52)	0.51(0.03,7.71)	5.22(0.50,54.70)	0.507	
Heavy drinker	400 (3.1%)	Ref	2.14 (0.34,13.33)	4.36 (1.00,18.97)	0.47 (0.05,4.15)	0.133	
Diabetes (*n*/%)							0.037
Yes	301 (2.3%)	Ref	0.48 (0.16,1.37)	0.98 (0.40,2.40)	2.09 (0.74,5.91)	0.605	
No	12,804 (97.7%)	Ref	1.01 (0.76,1.33)	0.93 (0.69,1.23)	0.67 (0.5,0.89)	0.004	
Coronary heart disease (*n*/%)							0.619
Yes	989 (7.5%)	Ref	1.29 (0.94,1.79)	0.93 (0.63,1.36)	0.78 (0.49,1.23)	0.103	
No	12,116 (92.5%)	Ref	0.93 (0.64,1.33)	0.84 (0.60,1.18)	0.67 (0.48,0.95)	0.035	

Adjusted for age, gender, race, PIR, marital status, education, BMI, smoking status, drinking status, diabetes, hypertension, hyperlipidemia, and coronary heart disease.

### 3.4 Dose–response relationship between HEI-2020 and HF risk

**Figure 3:**
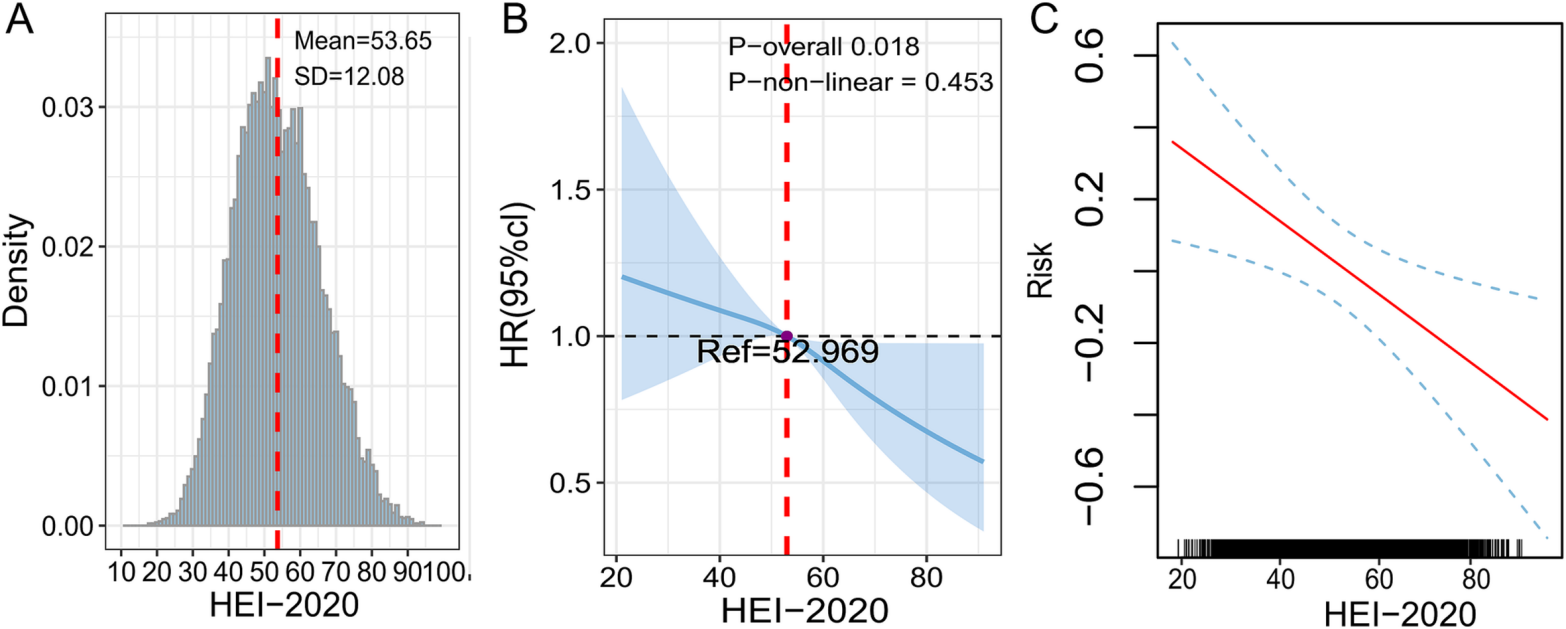
Dose–response relationship between HEI-2020 (in continuous form) and HF analyzed using RCS and GAM. **(A)** Density distribution of HEI-2020 scores. **(B)** RCS model. **(C)** GAM model. All models were adjusted for age, gender, race, PIR, marital status, education, BMI, smoking status, drinking status, diabetes, hypertension, hyperlipidemia, and coronary heart disease.

The RCS assisted in examining the dose–response relationship between HEI and HF, with results validated through the GAM. HEI-2020 scores in this study followed a normal distribution, with a mean and SD of 53.65 and 12.08, respectively, as shown in Figure 3A. A linear association was found between HEI-2020 and HF risk (*p-non-linear* = 0.453), as depicted in Figure 3B. The GAM results also demonstrated the same trend, as presented in Figure 3C.

### 3.5 Combined effects of 13 dietary components on HF risk

**Figure 4:**
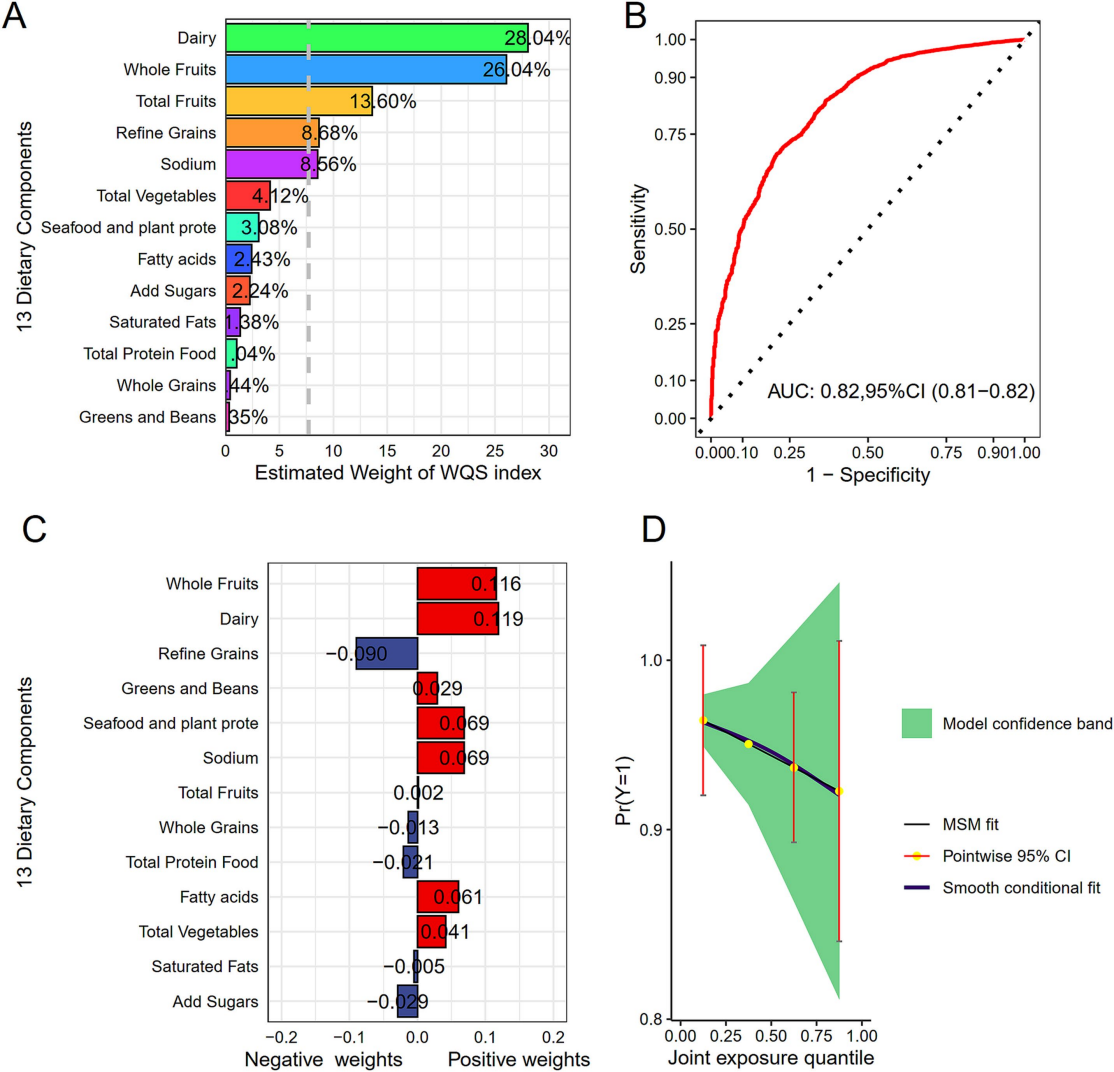
Display of combined effects. **(A)** WQS model for HF. **(B)** Contribution weights of dietary components in the WQS model for HF as indicated by AUC. **(C)** Contribution weights of dietary components in the Qgcomp model. **(D)** Visualization of trends in the Qgcomp model. All models included adjustment for age, gender, race, PIR, marital status, education, BMI, smoking status, drinking status, diabetes, hypertension, hyperlipidemia, as well as coronary heart disease.

We employed WQS regression model for assessing effects of 13 dietary components on HF risk mitigation, as displayed in Figures 4A,B. In Model 3, the WQS index of HEI-2020 (*OR* = 0.94, *95%CI*: 0.92–0.97) revealed a notable relation to a lowered HF risk. Specifically, dairy (28.04%), whole fruits (26.04%), and total fruits (13.60%) were found to be the most influential components, suggesting that they contributed the most to the reduction in HF risk. Finally, the WQS results was verified through the Qgcomp model. In Model 3, the findings from the Qgcomp model aligned with those from the WQS index (*OR* = 0.97, *95*%CI: 0.94–0.99). The dietary components that contributed most to HF risk reduction were whole fruits, dairy, and refined grains, as shown in Figures 4C,D.

## 4 Discussion

This study, involving seven cycles of NHANES (2005–2020) and 13,105 Americans aged 50 or above, elucidated the link of dietary quality evaluated through HEI-2020 to HF. Weighted logistic regression revealed a negative association between dietary quality and HF among individuals aged 50 or above. Subgroup analyses further confirmed this association across various subgroups, with particularly evident effects observed in current smokers and non-diabetic participants. Furthermore, weighted RCS demonstrated a linear connection between HEI-2020 and HF risk, indicating that the risk of HF decreased linearly as HEI-2020 scores increased within this age group. The GAM results corroborated the reliability of the weighted RCS findings. Finally, the contributions of 13 dietary components in HEI-2020 to mitigating HF risk were investigated through WQS and Qgcomp models. Whole fruits and dairy were proved to be the most contributory components, suggesting that these specific dietary components may have health benefits in reducing HF risk.

Previous research on dietary patterns has already proven the efficacy of nutritional strategies in preventing and treating cardiovascular diseases. A cohort study demonstrated that the DASH diet or substituting foods related to the DASH diet could offer protective effects against HF (15). Unlike the Western diet, the DASH diet features reduced sodium intake and increased consumption of foods abundant in vitamins, minerals, amino acids, and other bioactive substances in human cells (31). The DASH diet helps protect against HF via a combination of physiological mechanisms, including antioxidant, anti-inflammatory, antihypertensive, and anticoagulant effects (32, 33). Additionally, a prospective cohort study based on Swedish adults revealed benefits of the Mediterranean diet in lowering HF likelihood (*OR* = 0.79, *95% CI*: 0.68–0.93, *p* = 0.004) (34), showing a reduced HF risk among participants with higher adherence to this diet pattern compared with those with lower adherence (35). These results align with the findings of the present study. Furthermore, HEI is suitable for evaluating the dietary quality of Americans.

This study employed weighted RCS and GAM models to investigate the dose–response relation of HEI-2020 to HF risk in middle-aged and older individuals. The results demonstrated an adverse linear association between HEI and HF, which is consistent with the general understanding of health benefits of a healthy diet. Furthermore, this study replaced HEI-2020 scores with the combined effects of 13 dietary components to uncover the correlation of dietary quality with HF among older individuals, and the contribution of different components to reducing HF risk. In WQS and Qgcomp models, these 13 components positively influenced HF in older adults jointly. Increasing the intake of whole fruits and dairy appropriately was found to be particularly effective in mitigating the risk of HF in this population.

Fruit and dairy consumption is strongly linked to a decreased occurrence of cardiovascular diseases like atherosclerosis, hypertension, and HF (36, 37). Djoussé et al. (38) pointed out that healthy lifestyle factors, including maintaining a normal weight, abstaining from smoking, exercising frequently, consuming moderate amounts of alcohol, and eating fruits, vegetables, and cereals at breakfast, are strongly linked to a decreased lifetime risk of HF. Kakutani et al. (39) proved that more daily citrus fruit intake was linked to a reduced incidence of depression in chronic HF individuals. Vitamins C, carotenoids, and antioxidants in fruits contribute to cardiovascular health, which may indirectly mitigate the risk of HF (40, 41). Additionally, Zemel et al. (42) demonstrated that dairy consumption is beneficial for weight loss and provides vitamins and minerals that affect blood circulation. High levels of dairy intake may counteract HF-related brain damage by reducing weight, boosting blood flow, and ensuring sufficient oxygen delivery to the brain (43). Furthermore, lactose, probiotics, and lactic acid bacteria in dairy products stimulate intestinal microbial activity, promoting the production of butyrate. Moreover, fermented dairy products, such as butter, naturally contain small amounts of butyrate, which, upon absorption through the portal vein, interacts with various organs, demonstrating anti-inflammatory, anti-obesity, anti-angiogenic, and antioxidant functions. The foregoing biological activities suggest that butyrate may be therapeutic in cardiovascular disease prevention and treatment (44). Overall, these studies reflect the health benefits of fruit and dairy intake in cardiovascular diseases.

While the exact mechanisms linking a healthy diet to HF remain incompletely understood, several hypotheses have been proposed. First, regarding the anti-inflammatory/antioxidant effect, diets with high HEI-2020 scores typically include fruits, whole grains, vegetables, and nuts, which are foods with abundant antioxidants and anti-inflammatory components like vitamin C, polyphenols, and fiber. These components may slow the onset and progression of HF by lowering oxidative stress and inflammatory reactions (41). Second, in terms of improving blood glucose and lipid profiles, diets with high HEI-2020 scores often include dairy products rich in unsaturated fats, which help enhance cardiac function and myocardial metabolism, control blood glucose, and improve lipid profiles, thus lowering HF risk (37, 43). Lastly, considering the antihypertensive effect, healthy diets often involve abundant fruits, vegetables, grains, and dairy products, which all contain rich minerals like calcium, magnesium, phosphorus, and potassium. These nutrients are conducive to preventing HF by controlling blood pressure (45). Additionally, low sodium intake contributes to blood pressure reduction, as excessive sodium intake leads to fluid retention, increased blood pressure, and greater burden on heart, ultimately promoting the development of HF. In summary, HEI is associated with HF through multiple biological pathways, including anti-inflammatory/antioxidant effects, improved lipid/glucose profiles, and blood pressure reduction.

This study has several strengths. First, the utilization of a large, nationally representative NHANES dataset renders our conclusions applicable and representative. The results from subgroup analysis and interaction effects further support the stability and reliability of the findings. Additionally, the application of weighted RCS and GAM models demonstrated a linear association between HEI-2020 and HF. The WQS and Qgcomp models offered a visual representation of the contributions of 13 dietary components. However, several limitations must be acknowledged. First, HF and chronic disease covariates, including diabetes, hypertension, hyperlipidemia, and coronary heart disease, were obtained based on self-report questionnaires, which may lead to recall or reporting bias, potentially impacting the reliability of conclusions. Second, since NHANES was not specifically designed to focus on HF patients, it lacks data on the different causes of HF. Future studies should incorporate data on the specific causes of HF for clarifying the link of diet habits to HF subtypes. Third, given that the NHANES data used in this study spans the 2005–2020 survey period, there were differences in the collection of physical activity, which could influence the results. Fourth, this study is based on Americans, so the conclusions may not be directly applicable to populations in other countries or regions. Fifth, while we controlled for several potential confounding factors, it is impossible to eliminate the influence of all possible confounding factors. Finally, while a statistically significant difference was noted in HEI-2020 between HF and non-HF cohorts (51.64 vs. 53.77, *p* < 0.001), this difference merely reflects the overall difference in dietary patterns between the two groups. A single dietary factor cannot predict the occurrence of HF, as it is influenced by multiple factors, including genetic predisposition, metabolism, behavioral habits, and medical history. Additionally, this study was cross-sectional, and the causality between HEI and HF could not be validated. In summary, although we found a connection of HEI-2020 score with HF, the risk of HF cannot be predicted. Future longitudinal research, including prospective studies or randomized controlled trials, should elucidate the dynamic relation of HF to changes in HEI-2020 scores as well as to investigate the causal relationship between the two.

## 5 Conclusion

This study demonstrated a negative and linear dose–response relationship between dietary quality rated via HEI-2020 and HF among individuals aged 50 or above. The stability and reliability of this linear relationship were confirmed through subgroup analyses, and the WQS and Qgcomp models suggested the health benefits of whole fruits and dairy in mitigating HF risk. By highlighting this association, our study emphasizes the importance of adhering to the DGA in lowering HF risk in middle-aged and older individuals.

## Data Availability

The original contributions presented in the study are included in the article/Supplementary material, further inquiries can be directed to the corresponding author.

## Footnotes

^1^ https://cran.r-project.org/
