# Supplementary material for: Association between the healthy eating index 2020 and heart failure among the U.S. middle-aged and older adults from NHANES 2005–2020: a cross-sectional study
Source: Front Nutr. 2025 Jan 6;11:1496379. doi: 10.3389/fnut.2024.1496379 (PMC11743723; doi:10.3389/fnut.2024.1496379)
Supplement: Supplementary file 1 [file Table_1.docx]

Table S1 Baseline characteristics between participants included and not included in NHANES

| Characteristics (Weighted%) ^a^ | Include (n=13105) | Exclude (n=63391) | *p ^b^* |
| --- | --- | --- | --- |
| Median Age (IQR), years | 64.00 [57.00, 72.00] | 18.00 [8.00, 39.00] | <0.001 |
| Gender (n/%) |  |  | 0.010 |
| Male | 6305 (45.61%) | 31568 (49.84%) |  |
| Female | 6800 (54.39%) | 31823 (50.16%) |  |
| Race (n/%) |  |  | <0.001 |
| Mexican American | 1563 (4.15%) | 12821 (11.95%) |  |
| Other Hispanic | 1188 (3.68%) | 6383 (7.09%) |  |
| Non-Hispanic White | 6546 (78.21%) | 20901 (58.67%) |  |
| Non-Hispanic Black | 2872 (9.06%) | 15183 (13.02%) |  |
| Other-Race | 936 (4.90%) | 8103 (9.27%) |  |
| Education (n/%) |  |  | <0.001 |
| High school or below | 3262 (14.81%) | 24047 (29.73%) |  |
| High school or equivalent | 3164 (24.97%) | 6838 (15.07%) |  |
| College or above | 6679 (60.22%) | 15935 (39.80%) |  |
| Missing | 0 (0.00%) | 16571 (15.40%) |  |
| Marital status (n/%) |  |  | <0.001 |
| Married or living with partner | 7881 (66.39%) | 17709 (40.47%) |  |
| Divorced, separated, or widowed | 4327 (27.79%) | 5528 (9.58%) |  |
| Never married | 897 (5.82%) | 8749 (16.93%) |  |
| Missing | 0 (0.00%) | 31405 (33.03%) |  |
| PIR (n/%) |  |  | <0.001 |
| Low income (PIR<1) | 2161 (9.35%) | 16336 (17.33%) |  |
| Medium income (PIR≥1 and PIR<3) | 5621 (34.61%) | 23062 (33.67%) |  |
| High income(PIR≥3) | 5323 (56.05%) | 16691 (39.22%) |  |
| Missing | 0 (0.00%) | 7302 (9.79%) |  |
| Smoking status (n/%) |  |  | <0.001 |
| Never smoker | 6925 (54.16%) | 18253 (37.97%) |  |
| Former smoker | 4065 (30.63%) | 4502 (9.82%) |  |
| Current smoker | 2115 (15.21%) | 6385 (13.70%) |  |
| Missing | 0 (0.00%) | 34251 (38.51%) |  |
| Drinking status (n/%) |  |  | <0.001 |
| Never drinker | 3008 (18.21%) | 4840 (8.49%) |  |
| Former drinker | 2334 (14.92%) | 2744 (5.46%) |  |
| Mild drinker | 7032 (59.79%) | 16268 (40.73%) |  |
| Moderate drinker | 331 (3.06%) | 615 (1.67%) |  |
| Heavy drinker | 400 (4.03%) | 1008 (2.64%) |  |
| Missing | 0 (0.00%) | 37916 (41.01%) |  |
| Median BMI (IQR) | 28.60 [25.10, 32.90] | 23.80 [18.60, 29.40] | <0.001 |
| Diabetes (n/%) | 301 (1.76%) | 333 (0.47%) | <0.001 |
| Hypertension (n/%) | 1867 (13.03%) | 3452 (5.51%) | <0.001 |
| Hyperlipidemia (n/%) | 1073 (7.27%) | 2717 (4.43%) | <0.001 |
| Coronary heart disease (n/%) | 989 (6.83%) | 813 (1.34%) | <0.001 |
| Heart failure (n/%) | 735 (4.37%) | 780 (1.08%) | <0.001 |

a The number of participants is unweighted. All percentage estimates are weighted.

b P value was based on χ2 or analysis of variance or Kruskal-Wallis rank sum test where appropriate.
