# Supplementary material for: Association between the healthy eating index 2020 and heart failure among the U.S. middle-aged and older adults from NHANES 2005–2020: a cross-sectional study
Source: Front Nutr. 2025 Jan 6;11:1496379. doi: 10.3389/fnut.2024.1496379 (PMC11743723; doi:10.3389/fnut.2024.1496379)
Supplement: Supplementary file 2 [file Table_2.docx]

**Table S2** The characteristics by quartile of the HEI-2020.

| Characteristics (Weighted%) ^a^ | HEI-2020 Score | | | | | *p^b^* |
| --- | --- | --- | --- | --- | --- | --- |
|  | Total (n=13105) | Q1 (<44.86, n=3277) | Q2 (44.86-52.97, n=3276) | Q3 (52.97-61.73, n=3276) | Q4 (≥61.73,n=3276) |  |
| Median Age (IQR), years | 64.00 [57.00, 72.00] | 62.00 [55.00, 70.00] | 63.00 [56.00, 72.00] | 64.00 [57.00, 73.00] | 65.00 [58.00, 73.00] | <0.001 |
| Gender (n/%) |  |  |  |  |  |  |
| Male | 6305 (45.63%) | 1763 (51.43%) | 1638 (48.37%) | 1508 (42.15%) | 1396 (41.07%) | <0.001 |
| Female | 6800 (54.37%) | 1514 (48.57%) | 1638 (51.63%) | 1768 (57.85%) | 1880 (58.93%) |  |
| Race (n/%) |  |  |  |  |  | <0.001 |
| Mexican American | 1563 (4.26%) | 357 (4.25%) | 419 (4.58%) | 432 (4.70%) | 355 (3.53%) |  |
| Other Hispanic | 1188 (3.71%) | 230 (3.22%) | 282 (3.46%) | 319 (3.76%) | 357 (4.33%) |  |
| Non-Hispanic White | 6546 (77.52%) | 1632 (75.88%) | 1631 (78.06%) | 164 2(78.04%) | 1641 (78.00%) |  |
| Non-Hispanic Black | 2872 (9.14%) | 886 (12.11%) | 781 (10.06%) | 651 (7.99%) | 554 (6.64%) |  |
| Other-Race | 936 (5.37%) | 172 (4.54%) | 163 (3.84%) | 232 (5.50%) | 369 (7.50%) |  |
| Education (n/%) |  |  |  |  |  | <0.001 |
| High school or below | 3262 (14.78%) | 946 (18.88%) | 903 (16.58%) | 800 (13.71%) | 613 (10.35%) |  |
| High school or equivalent | 3164 (25.18%) | 961 (32.94%) | 858 (29.02%) | 752 (21.91%) | 593 (17.55%) |  |
| College or above | 6679 (60.04%) | 1370 (48.18%) | 1515 (54.41%) | 1724 (64.38%) | 2070 (72.11%) |  |
| Marital status (n/%) |  |  |  |  |  | <0.001 |
| Married or living with partner | 7881 (65.92%) | 1856 (61.71%) | 1964 (66.13%) | 1993 (66.17%) | 2068 (69.35%) |  |
| Divorced, separated, or widowed | 4327 (28.24%) | 1132 (30.86%) | 1079 (28.09%) | 1075 (28.33%) | 1041 (25.90%) |  |
| Never married | 897 (5.84%) | 289 (7.43%) | 233 (5.79%) | 208 (5.50%) | 167 (4.75%) |  |
| PIR (n/%) |  |  |  |  |  |  |
| Low income (PIR<1) | 2161 (9.66%) | 686 (13.28%) | 606 (11.36%) | 494 (8.00%) | 375(6.32%) | <0.001 |
| Medium income (PIR≥1 and PIR<3) | 5621 (34.78%) | 1577 (41.08%) | 1430 (35.75%) | 1369 (32.97%) | 1245 (29.86%) |  |
| High income(PIR≥3) | 5323 (55.56%) | 1014 (45.64%) | 1240 (52.90%) | 1413 (59.04%) | 1656 (63.82%) |  |
| Smoking status (n/%) |  |  |  |  |  | <0.001 |
| Never smoker | 6925 (53.74%) | 1414 (45.80%) | 1646 (49.44%) | 1823 (57.13%) | 2042 (61.86%) |  |
| Former smoker | 4065 (30.65%) | 959 (26.68%) | 994 (31.73%) | 1074 (32.50%) | 1038 (31.45%) |  |
| Current smoker | 2115 (15.61%) | 904 (27.52%) | 636 (18.83%) | 379 (10.37%) | 196 (6.70%) |  |
| Drinking status (n/%) |  |  |  |  |  | <0.001 |
| Never drinker | 3008 (18.23%) | 688 (17.75%) | 739 (17.69%) | 772 (17.99%) | 809 (19.43%) |  |
| Former drinker | 2334 (14.57%) | 737 (19.17%) | 611 (15.60%) | 528 (13.45%) | 458 (10.47%) |  |
| Mild drinker | 7032 (59.83%) | 1707 (57.41%) | 1710 (59.31%) | 1769 (60.57%) | 1846 (61.82%) |  |
| Moderate drinker | 331 (3.25%) | 65 (2.47%) | 97 (3.39%) | 95 (3.86%) | 74 (3.27%) |  |
| Heavy drinker | 400 ( 4.11%) | 80 (3.21%) | 119 (4.02%) | 112 (4.13%) | 89 (5.02%) |  |
| BMI (Mean±SD) | 29.6±6.65 | 30.45±7.28 | 30.08±6.86 | 29.43±6.30 | 28.45±5.92 | <0.001 |
| Diabetes (n/%) | 301 (1.72%) | 78 (2.21%) | 91 (2.14%) | 79 (1.47%) | 53 (1.13%) | 0.015 |
| Hypertension (n/%) | 1867 (13.31%) | 514 (14.06%) | 488 (14.56%) | 448 (12.13%) | 417 (12.57%) | 0.003 |
| Hyperlipidemia (n/%) | 1073 (7.50%) | 330 (10.45%) | 278 (8.07%) | 251 (6.42%) | 214 (5.30%) | <0.001 |
| Coronary heart disease (n/%) | 989 (6.84%) | 258 (7.14%) | 223 (5.56%) | 270 (7.83%) | 238 (6.84%) | 0.127 |
| Heart failure(n/%) | 735 (4.44%) | 222 (4.97%) | 188 (4.81%) | 185 (4.53%) | 140 (3.50%) | <0.001 |

a The number of participants is unweighted. All percentage estimates are weighted.

b P value was based on χ2 or analysis of variance or Kruskal-Wallis rank sum test where appropriate.
